# Cost-effectiveness of adjuvant paclitaxel and trastuzumab for early-stage node-negative, HER2-positive breast cancer

**DOI:** 10.1371/journal.pone.0217778

**Published:** 2019-06-05

**Authors:** Ali Hajjar, Mehmet A. Ergun, Oguzhan Alagoz, Murtuza Rampurwala

**Affiliations:** 1 Department of Industrial & Systems Engineering, University of Wisconsin-Madison, Madison, Wisconsin, United States of America; 2 Industrial Engineering Department, Istanbul Şehir University, Istanbul, Turkey; 3 Section of Hematology/Oncology, University of Chicago, Chicago, Illinois, United States of America; Harran Universitesi, TURKEY

## Abstract

**Objectives:**

Adjuvant paclitaxel and trastuzumab has been shown to be an effective regimen with low risk of cancer recurrence and treatment-related toxicities in early-stage node-negative, HER2-positive breast cancer. We investigated the cost-effectiveness of this regimen.

**Methods:**

A Markov-based microsimulation model with six health states is used to simulate four adjuvant therapy options for women with early-stage node-negative, HER2-positive breast cancer at different age groups. The four treatment arms are 1) adjuvant paclitaxel and trastuzumab (TH), 2) doxorubicin, cyclophosphamide, paclitaxel and trastuzumab (ACTH), 3) docetaxel, carboplatin and trastuzumab (TCH), and 4) no adjuvant trastuzumab (NT). Data from randomized trials were used to estimate treatment efficacy. Societal perspective was used in this cost-effectiveness analysis. Costs were measured in 2016 US dollars (US$) and quality-adjusted life-years (QALYs) was used for health outcomes. Sensitivity analyses were performed to evaluate the impact of uncertainty in parameter estimation.

**Results:**

We found that 40-year-old women undergoing TH treatment would have an average of 16.17 QALYs for the cost of $178,650 when lifetime horizon is used. Compared to NT, TH has incremental cost-effectiveness ratios ranged from $10,584 (ages 40–49) to $84,981 (age 80+) per additional QALYs. The sensitivity analysis showed that TH is cheaper and leads to higher QALYs compared to both ACTH and TCH for all age groups and time horizons.

**Conclusions:**

TH is cost-effective for all age groups in the base case scenario and in the sensitivity analysis. In order to reduce the parameter uncertainty, clinical trials with longer follow-up times are needed.

## 1 Introduction

Breast cancer remains the most common cancer among the US women with an estimated 252,710 diagnoses and over 40,000 deaths in 2017 [1]. Human epidermal growth factor receptor type 2 (HER2) is amplified and overexpressed in 15–20% of breast cancers and historically has been associated with inferior disease-related outcomes [2,3]. Trastuzumab, a humanized monoclonal antibody to the HER2 protein, in combination with chemotherapy has been shown to improve disease outcomes and survival in the adjuvant setting with early-stage HER2-positive breast cancer [4–6]. These pivotal clinical trials established doxorubicin, cyclophosphamide, paclitaxel and trastuzumab (ACTH) and non-anthracycline based regimens such as docetaxel, carboplatin and trastuzumab (TCH) as standard of care adjuvant treatment for HER2-positive breast cancer.

Because the majority of patients enrolled in the early trastuzumab studies had higher-risk disease with larger, node-positive tumors, and with concerns of toxicities from adjuvant chemotherapy, there has been increasing interest in de-escalation of therapy for low-risk disease. Tolaney and colleagues recently reported disease-related outcomes and low toxicities with a novel paclitaxel and trastuzumab (TH) adjuvant therapy regimen in early-stage node-negative tumors with risk of disease recurrence of less than 2% at 3 years [7]. Despite the short duration of follow-up, based on promising reported outcomes and tolerability, TH is being increasingly utilized in clinical practice for adjuvant treatment of low-risk HER2-positive disease.

With the clinical benefit of adjuvant trastuzumab alongside the escalating costs of cancer treatments, we sought to assess the cost-effectiveness of the TH regimen as compared to standard of care, ACTH and TCH regimens for small, node-negative HER2-positive breast cancer in the United States using a Markov modeling approach. We also included an arm of no adjuvant trastuzumab (NT), based on retrospective reports of outcomes reported in this context [8,9].

## 2 Methods

### 2.1 Study design

**Fig 1:**
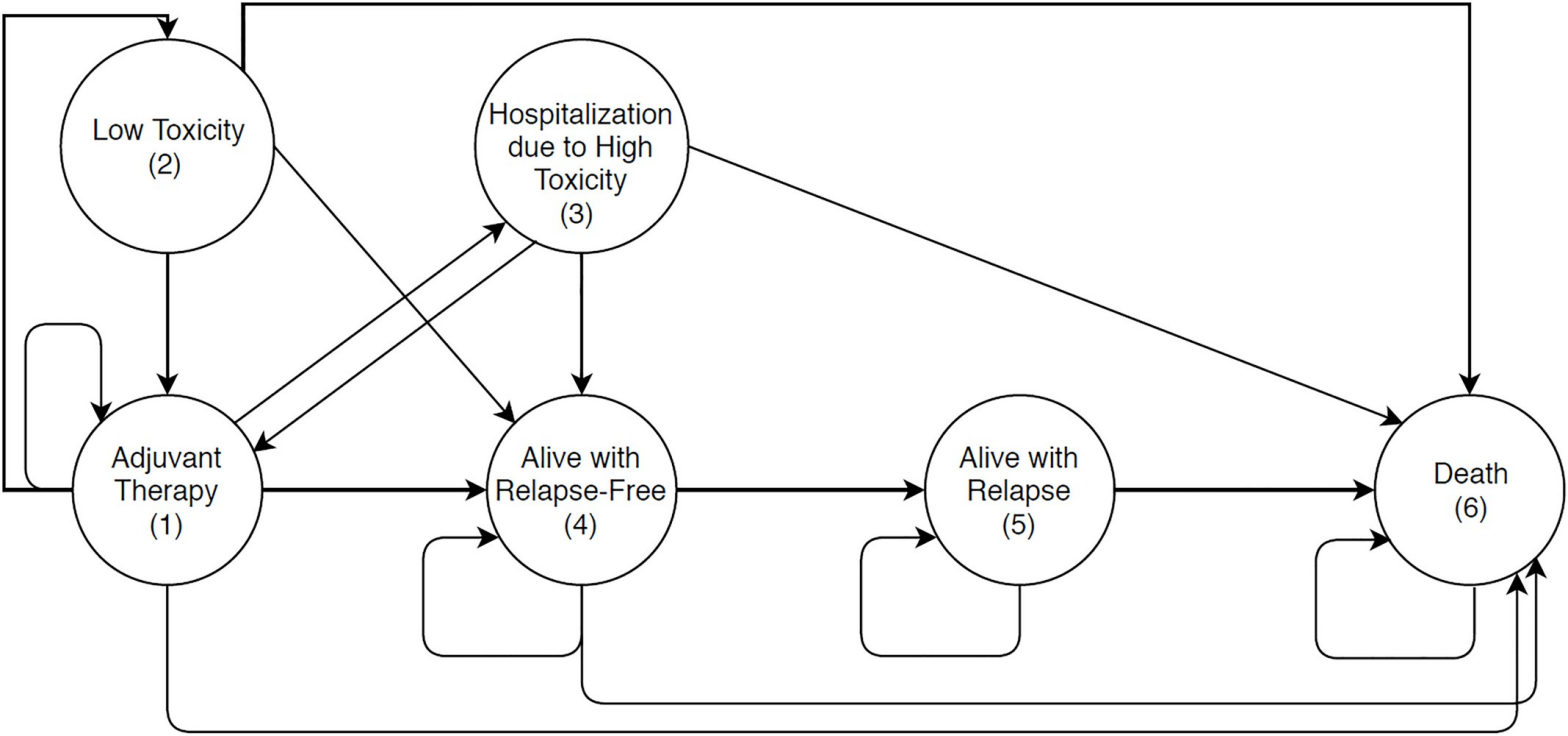
State-transition Markov model represents breast cancer treatment and follow-up states. This figure represents the conceptual model used in this study.

A Markov state-transition microsimulation model was developed in MATLAB 2016 software to evaluate the cost-effectiveness of four adjuvant treatment strategies for early-stage node-negative, HER2-positive breast cancer (Fig 1; see also S1 and S2 Figs). The Markov model uses a cycle length of one month to simulate 10,000 patients in each arm representing different treatment strategies. As per the study by Kurian et al.[10], the model evaluated the lifetime health outcomes beyond the 4 & 5 years median follow-up data available from the randomized clinical trials [6,7] to estimate the long-term impact on health outcomes, treatments’ costs and quality of life. This study follows the Consolidated Health Economic Evaluation Reporting Standards (CHEERS) [11], the CHEERS checklist associated with this study is included in the Supplemental Materials (S1 Checklist).

We presented the results in terms of incremental cost-effectiveness ratios (ICERs) using two different effectiveness measures: cost per quality-adjusted life-years (QALYs) gained and cost per life-years (LYs) gained. Following the recommendations of the Panel on Cost-Effectiveness in Health and Medicine, we performed our analysis using the societal perspective and discounted both future costs and future QALYs at 3% in the base case analysis [12]. We preferred the societal perspective since it reflects the viewpoint of the various decision makers considering the broad allocation of resources across the population and allows the comparison of the present study to other programs used for other diseases and health conditions. [12] Furthermore, because our study could be used by various decision makers such as the insurance companies, individual payers, and providers, limiting the perspective to only one type of decision makers would our results less useful for others.

Our model considers five age groups (40–49, 50–59, 60–69, 70–79 and 80+ year-old women) with early-stage node-negative HER2-positive breast cancer. Age groups 40–49 and 50–59 represent the median age group of the participants in the addressed randomized trials [6,7]. We used age-based mortality rates to evaluate the cost-effectiveness for age groups, 60–69, 70–79 and 80+ year-old women. Assuming the relapse rate will decline beyond the median follow-up period [10], we extrapolate the relapse rates reported in [6,7] for the subsequent years with a similar rate.

### 2.2 Treatment strategies

This cost-effectiveness analysis evaluates and compares the clinical and cost outcomes of four alternative adjuvant therapies. The first arm represents the no adjuvant trastuzumab (NT) which is based on the control arms of the NSABPB-31 and NCCTG9831 clinical trials [4]. The second arm simulates the TH regimen [7], where paclitaxel is administered at 80 mg/square meter of body-surface area weekly for three months with a trastuzumab dose of 4 mg/kg of body weight on day 1 and then, 2 mg of intravenous trastuzumab per kilogram weekly, for a full year.

ACTH and TCH regimens were considered as comparative programs [6]. These two regimens are commonly used in practice and the clinical trials on these therapies included early-stage node-negative breast cancer patients. In the third arm, patients receive ACTH where cyclophosphamide and doxorubicin are administrated at 600 mg/m2 and 60 mg/m2 respectively for 4 cycles every 3 weeks. Then, patients receive docetaxel 100 mg/m2 for 4 doses every 3 weeks and 52 weeks of trastuzumab [6]. In the fourth arm, patients receive TCH where carboplatin is administered at 6 mg per milliliter per minute and docetaxel at 75 mg/m2 for 6 cycles and every 3 weeks along with trastuzumab concurrently. Then, patients receive trastuzumab alone to complete a year of treatment [6].

### 2.3 Model structure

The Markov model is essentially divided into two sub-models: adjuvant chemotherapy sub-model and follow-up sub-model (Fig 1). Markov sub-model for the adjuvant chemotherapy consists of the following 5 states: adjuvant therapy (state 1), low toxicity (state 2), hospitalization due to high toxicity (state 3), alive with relapse-free (state 4), and death due to any cause (state 6). All patients start the simulation at the adjuvant therapy state (state 1) and transitions among various states occur according to a Markov process. There are three levels of toxicity due to adjuvant chemotherapy based on the grading system: well, low toxicity, and high toxicity. No adverse effects (grade 0) are represented by “Well” when patients continue to undergo treatment, minimal or moderate adverse events (grade 1 and 2) are represented by ‘‘low toxicity,” and severe or catastrophic adverse events (grade 3 and 4) are represented by ‘‘high toxicity”[13]. If a patient receives adjuvant therapy with trastuzumab, the adjuvant therapy sub-model runs for 12 months; and if she receives no adjuvant trastuzumab (NT), the sub-model runs for 6 months where transitions occur on a monthly basis.

Once a patient completes adjuvant therapy or quits adjuvant therapy prematurely, she moves directly to the follow-up sub-model and starts in the “alive with relapse-free” state (state 4). This sub-model consists of three states. Two of these states are identical to those in the adjuvant chemotherapy sub-model (states 4 and 6). The third state is “alive with relapse” (state 5). Upon a breast cancer recurrence or a new primary breast cancer event, the patient undergoes the same treatment she had in the adjuvant chemotherapy sub-model one more time and remains at the “alive with relapse” state until death. This assumption is made only for local recurrence or a new primary breast cancer which would be treated with curative intent with adjuvant therapy. Due to limited data, we assumed similar relapse probability for all age groups in each of the adjuvant therapy arms. Patients are followed until they die (i.e., the time horizon of the study is the lifetime of the patient).

### 2.4 Transition probabilities

We adjusted all rates to estimate the monthly health-state transition probabilities (Table 1). We used age-based mortality rates to estimate the monthly death probability from the National Center for Vital Statistics [14] since no patients following TH regimen died due to breast cancer. For the NT arm, we estimated breast cancer recurrence rates based on the study by Kurian et al.[10], which are based on the control arms of the NSABPB-31 and NCCTG9831 clinical trials [4]. In all trastuzumab arms, we estimated the efficacy and safety input parameters for each regimen using the most recent randomized clinical trials [6,7]. Beyond the median follow-up period of these trials, we considered that the relapse rate would decline as assumed by Kurian et al. [10]. We considered distant, regional, and local recurrence as well as new contralateral primary breast cancer in estimating the monthly rate of relapse. For the ACTH and TCH therapies, we estimated the relapse rate based only on the group of patients with early-stage node-negative breast tumors to ensure a fair comparison. Additionally, we assumed that if a patient had to quit the therapy for any reason, her monthly relapse rate would increase linearly to 0.0126 [8]. We also assumed no breast cancer relapse during the treatment period.

**Table 1:** Base case transition probabilities.

Arm	Parameter Name	Age	Base Case Monthly Probability (Mean)	Range / SD	Reference
Control arm (NT)	Death from all states, years 1–2 (except alive with relapse state)	40–49	0.00040	Not varied	[10]
		50–59 ^e^	0.00102	Not varied	[10]
		60–69 ^e^	0.00236	Not varied	[10]
		70–79 ^e^	0.00548	Not varied	[10]
		80 and over ^e^	0.01485	Not varied	[10]
	Death from all states, years >2 (except alive with relapse state) (age-specific mortality)	40–49	0.00034	Not varied	[10,14]
		50–59	0.00078	Not varied	[10,14]
		60–69	0.00161	Not varied	[10,14]
		70–79	0.00403	Not varied	[10,14]
		80 and over	0.01485	Not varied	[10,14]
	Relapse, years 1–2 ^a^^,^^d^	All age groups	0.007	20% +/-	[10]
	Relapse, years 3–4 ^a^^,^^d^		0.0085	20% +/-	[10]
	Relapse, years 5–8 ^a^^,^^d^		0.0045	20% +/-	[10]
	Relapse, years 9–10 ^a^^,^^d^		0.0032	20% +/-	[10]
	Relapse, years ≥11 ^a^^,^^d^		0.0016	20% +/-	[10]
	Death from relapse		0.02734	50% +/-	[10]
TH	Death from all states (except alive with relapse state) (age-specific mortality)	40–49	0.00034	Not varied	[7,14]
		50–59	0.00078	Not varied	[7,14]
		60–69	0.00161	Not varied	[7,14]
		70–79	0.00403	Not varied	[7,14]
		80 and over	0.01485	Not varied	[7,14]
	Relapse, years 1–2 ^a^^,^^d^	All age groups	0.00042	20% +/-	[7,10]
	Relapse, years 3–4 ^a^^,^^d^		0.00051	20% +/-	[7,10]
	Relapse, years 5–8 ^a^^,^^d^		0.00027	20% +/-	[7,10]
	Relapse, years 9–10 ^a^^,^^d^		0.00019	20% +/-	[7,10]
	Relapse, years ≥11 ^a^^,^^d^		0.00010	20% +/-	[7,10]
	Death from relapse		0.02734	50% +/-	[10]
	High toxicity event		0.00170	[.00021 - .003]	[7]
	Quit treatment due to high toxicity ^b^		0.00340	10% +/-	[7]
	Low toxicity event ^c^		0.00761	[.0047 -.0165]	[7]
	Quit treatment due to low toxicity ^b^		0.00148	10% +/-	[7]
ACTH	Death from all states (except alive with relapse state) (age-specific mortality)	40–49	0.00034	Not varied	[14]
		50–59	0.00078	Not varied	[14]
		60–69	0.00161	Not varied	[14]
		70–79	0.00403	Not varied	[14]
		80 and over	0.01485	Not varied	[14]
	Relapse, years 1–2 ^a^^,^^d^	All age groups	0.00094	20% +/-	[6,10]
	Relapse, years 3–4 ^a^^,^^d^		0.00114	20% +/-	[6,10]
	Relapse, years 5–8 ^a^^,^^d^		0.00060	20% +/-	[6,10]
	Relapse, years 9–10 ^a^^,^^d^		0.00043	20% +/-	[6,10]
	Relapse, years ≥11 ^a^^,^^d^		0.00021	20% +/-	[6,10]
	Death from relapse		0.02734	50% +/-	[10]
	High toxicity event		0.00980	[.000078 - .058]	[6]
	Quit treatment due to high toxicity ^b^		0.00340	10% +/-	[7]
	Low toxicity event ^c^		0.04250	[.0017 - .247]	[6]
	Quit treatment due to low toxicity ^b^		0.00148	10% +/-	[7]
TCH	Death from all states (except alive with relapse state) (age-specific mortality)	40–49	0.00034	Not varied	[14]
		50–59	0.00078	Not varied	[14]
		60–69	0.00161	Not varied	[14]
		70–79	0.00403	Not varied	[14]
		80 and over	0.01485	Not varied	[14]
	Relapse, years 1–2 ^a^^,^^d^	All age groups	0.00131	20% +/-	[6,10]
	Relapse, years 3–4 ^a^^,^^d^		0.00159	20% +/-	[6,10]
	Relapse, years 5–8 ^a^^,^^d^		0.00084	20% +/-	[6,10]
	Relapse, years 9–10 ^a^^,^^d^		0.00060	20% +/-	[6,10]
	Relapse, years ≥11 ^a^^,^^d^		0.00030	20% +/-	[6,10]
	Death from relapse		0.02734	50% +/-	[10]
	High toxicity event		0.00920	[.000079 - .053]	[6]
	Quit treatment due to high toxicity ^b^		0.00340	10% +/-	[7]
	Low toxicity event ^c^		0.03983	[.0018 - .230]	[6]
	Quit treatment due to low toxicity ^b^		0.00148	10% +/-	[7]

^a^ Considering the ratio of the NT arm.

^b^ We assumed the same probability as in the TH arm given the absence of data—applied for all trastuzumab arms.

^c^ We used the ratio between high and low toxicity in the TH to estimate the low toxicity ratio here.

^d^ Probability of relapse increases linearly if a patient quits the treatment for any reason / toxicity.

^e^ We used age-specific mortality ratio to come up with probability for the different age groups.

Abbreviations: ACTH, doxorubicin, cyclophosphamide, paclitaxel and trastuzumab regimen; NT, no adjuvant trastuzumab regimen; TCH, docetaxel, carboplatin and trastuzumab regimen; TH, adjuvant paclitaxel and trastuzumab regimen.

### 2.5 Treatment-related toxicity

In this study, we considered toxicity events resulted from using trastuzumab in the adjuvant therapy. Low and high toxicity rates were derived from the respective randomized trials [6,7]. We assumed that patients quit the treatment after one month if they did not recover from a toxicity event as in the trial protocol of Tolaney et al. [7]. We assumed that 70% of the patients who quit the treatment quit due to high toxicity and 30% of them quit due to low toxicity as reported by [4].

### 2.6 Utilities

We used latest published studies to estimate the quality-of-life for the adjuvant therapies, low and high toxicities, and breast cancer relapse (Table 2). For low toxicity events, we estimated the utility based on the utility of chemotherapy-induced nausea and vomiting (0.85) for patients who are undergoing adjuvant therapy with trastuzumab. For hospitalization due to high toxicity, we used the mean utilities of patients with febrile neutropenia (0.47), congestive heart failure (0.87), cardiac toxicity with symptoms (0.64) and acute myeloid leukaemia and/or myelodysplastic syndrome (0.26). On the other hand, we applied a utility of (0.78) for the ACTH adjuvant therapy and a utility of (0.81) for the NT therapy. Due to limited data, we assumed that TCH adjuvant therapy has similar utility as ACTH and we assumed a mid-point utility of (0.795) for the TH regimen since it is expected to cause less toxicity compared to both TCH and ACTH [6,7]. We did a sensitivity analysis on this parameter.

**Table 2:** Base case utilities and costs for various health states, treatments, breast cancer relapse, and toxicity levels.

Treatment Arm	Parameter Name	Age	Base Case (Mean)	Range / SD		Reference
**Health state utilities**			**Monthly utility**			
NT	Therapy	All age groups	0.810	[.75 - .95]		[10,15]
All trastuzumab arms	Utility due to high toxicity	All age groups	0.560	[.26 - .87]		[10,16]
	Utility due to low toxicity		0.850	.04	Beta	
TH	Adjuvant therapy	All age groups	0.795	[.75 - .95]		Assumed based on [6,10]
ACTH and TCH ^a^	Adjuvant therapy	All age groups	0.780	[.6 -.95]		[10,15]
All arms	Patient utility before starting the treatment	40–49	0.84	0.002	Beta	[16]
		50–59	0.83	0.003	Beta	
		60–69	0.83	0.003	Beta	
		70–79	0.81	0.004	Beta	
		80 and over	0.78	0.006	Beta	
	Utility of relapse	All age groups	0.55	20% +/-		[10]
	Utility after therapy (relapse-free)		0.90	[.85 - .95]		[15]
**Cost**			**Monthly cost** **2016 US dollars**			
NT	Treatment cost ^b^^,^^c^	All age groups	$5,896.59	20% +/-		[10,15,17]
TH	Treatment cost ^b^^,^^c^	All age groups	$7,812.34	20% +/-		[10,15,17]
TCH	Treatment cost ^b^^,^^c^	All age groups	$9,404.05	20% +/-		[10,15,17]
ACTH	Treatment cost ^b^^,^^c^	All age groups	$11,452.91	20% +/-		[10,15,17]
All trastuzumab arms	High toxicity	All age groups	$2,730.0	[2068–5517]		[15]
	Low toxicity		$212.00	20% +/-		[10]
All arms	Relapse-free state	40–49	$286.00	Not varied		[10]
		50–59	$311.50	Not varied		
		60–69	$382.50	Not varied		
		70 and over	$425.50	Not varied		
	Death	All age groups	$10,000.00	[5000–20,000]		[15]

^a^ Assumed to be similar to the ACTH.

^b^ The recurrence cost is similar.

^c^ Includes drug, infusion, 15-minute oncologist visit monthly, cardiac monitoring, and costs of time lost from work and travel cost.

Abbreviations: ACTH, doxorubicin, cyclophosphamide, paclitaxel and trastuzumab regimen; NT, no adjuvant trastuzumab regimen; TCH, docetaxel, carboplatin and trastuzumab regimen; TH, adjuvant paclitaxel and trastuzumab regimen.

### 2.7 Resource use and costs

Using the medical care component of the consumer price index, we converted all costs into 2016 US dollars (Table 2). For each therapy, we considered the costs of monthly oncologist visit, drug infusion, toxicity monitoring, as well as costs due to being out of work and travel-related costs. We estimated these costs from publications which incorporated data from the Centers for Medicare & Medicaid Services and the US Bureau of Labor Statistics [10,15]. We estimated the total costs of the adjuvant chemotherapies using the Red Book average wholesale drug prices as per clinical trials’ protocol. [6,7,15,17]. Based on the latest Common Terminology Criteria for Adverse Events (CTCAE) v5.0, we estimated the cost of a hospitalization due to high toxicity events based on grade 3–4 adverse events [13]. Thus, the estimated cost of hospitalization due to high toxicity events accounts for the costs related to cardiotoxicity events [15]. Furthermore, we used grade 1–2 adverse events to estimate the cost of low toxicity events as they are minimal, asymptomatic, and do not impair daily living activities [10,13].

### 2.8 Validation and sensitivity analysis

We utilized several methods for validation. First, we ensured the face-validity of the model by consulting our clinician co-author to share his clinical expertise on the model’s structure, assumptions, and the input parameters. Additionally, all health-related parameters were derived directly from the relevant clinical trials. Finally, a cross-validation was performed by comparing our model’s results for the no adjuvant trastuzumab (NT) treatment strategy with those of the study by Kurian et al. [10].

We utilized different methods to perform sensitivity analyses in order to test the robustness of the base case results. We conducted one-way and probabilistic sensitivity analyses to account for uncertainty in model parameters. We varied the model parameters based on the distributions as reported in Tables 1 and 2. For parameters with uncertainty reported in the form of a range, we assumed triangular distribution by using the mean value as the mode. We used common random numbers to compare different treatment alternatives [18]. We used $50,000/QALYs as the willingness-to-pay (WTP) threshold [19].

Furthermore, we evaluated the cost-effectiveness for different age groups in 40 to 80 years old. We determined the age-based ICER assuming all parameters are kept constant except for the mortality rates, patient utility without the treatment, and the monthly cost of being at the “alive with relapse-free” state. We also evaluated the cost-effectiveness of considered treatments when different time horizons are used.

## 3 Results

### 3.1 Base case

The results of the base case analysis over a lifetime horizon for age group 40–49 years (Table 3) show that the no adjuvant trastuzumab (NT) arm yields 9.57 QALYs and 18.87 LYs at a cost of $108,821, the paclitaxel and trastuzumab regimen yields 16.17 QALYs (and 33.77 LYs) at a cost of $178,650, the ACTH regimen yields 15.46 QALYs (and 32.25 LYs) at a cost of $221,974, and the TCH regimen yields 15.02 QALYs (and 31.23 LYs) at a cost of $198,473. Compared to the NT arm, the use of the TH regimen would incur an additional cost of $69,829 for an expected gain of 6.6 QALYs (and 14.9 LYs). Hence, the incremental cost per QALY gained is $10,584. Furthermore, the TH regimen is cheaper and leads to higher QALYs compared to both ACTH and TCH regimens.

**Table 3:** Base case results for the 40–49 age group when lifetime horizon is used.

**Adjuvant Treatment**	**Total Cost** **(2016 USD)**	**QALYs**	**Incremental costs** ^**a**^	**Incremental QALYs** ^**a**^	**ICER**
(Base case)					
NT	$ 108,821	9.57	$ -69,829	-6.6	—
TH	$ 178,650	16.17	—	—	$ 10,584 / QALY ^**b**^
ACTH	$ 221,974	15.46	$ 43,324	-0.71	N/A ^**a**^
TCH	$ 198,473	15.02	$ 19,823	-1.15	N/A ^**a**^
(Without adjustment for quality of life)	**Total Cost**	**LYs**	**Incremental costs**	**Incremental LYs**	**ICER**
NT	$ 108,821	18.87	$ -69,829	-14.9	—
TH	$ 178,650	33.77	—	—	$ 4,688 / LY ^**b**^
ACTH	$ 221,974	32.25	$ 43,324	-1.52	N/A ^**a**^
TCH	$ 198,473	31.23	$ 19,823	-2.54	N/A ^**a**^

^a^ Not applicable.

^b^ Relative to NT.

Abbreviations: ACTH, doxorubicin, cyclophosphamide, paclitaxel and trastuzumab regimen; ICER, incremental cost-effectiveness ratio; NT, no adjuvant trastuzumab regimen; QALY, quality-adjusted life year; TCH, docetaxel, carboplatin and trastuzumab regimen; TH, adjuvant paclitaxel and trastuzumab regimen.

### 3.2 Impact of age and time horizon

**Fig 2:**
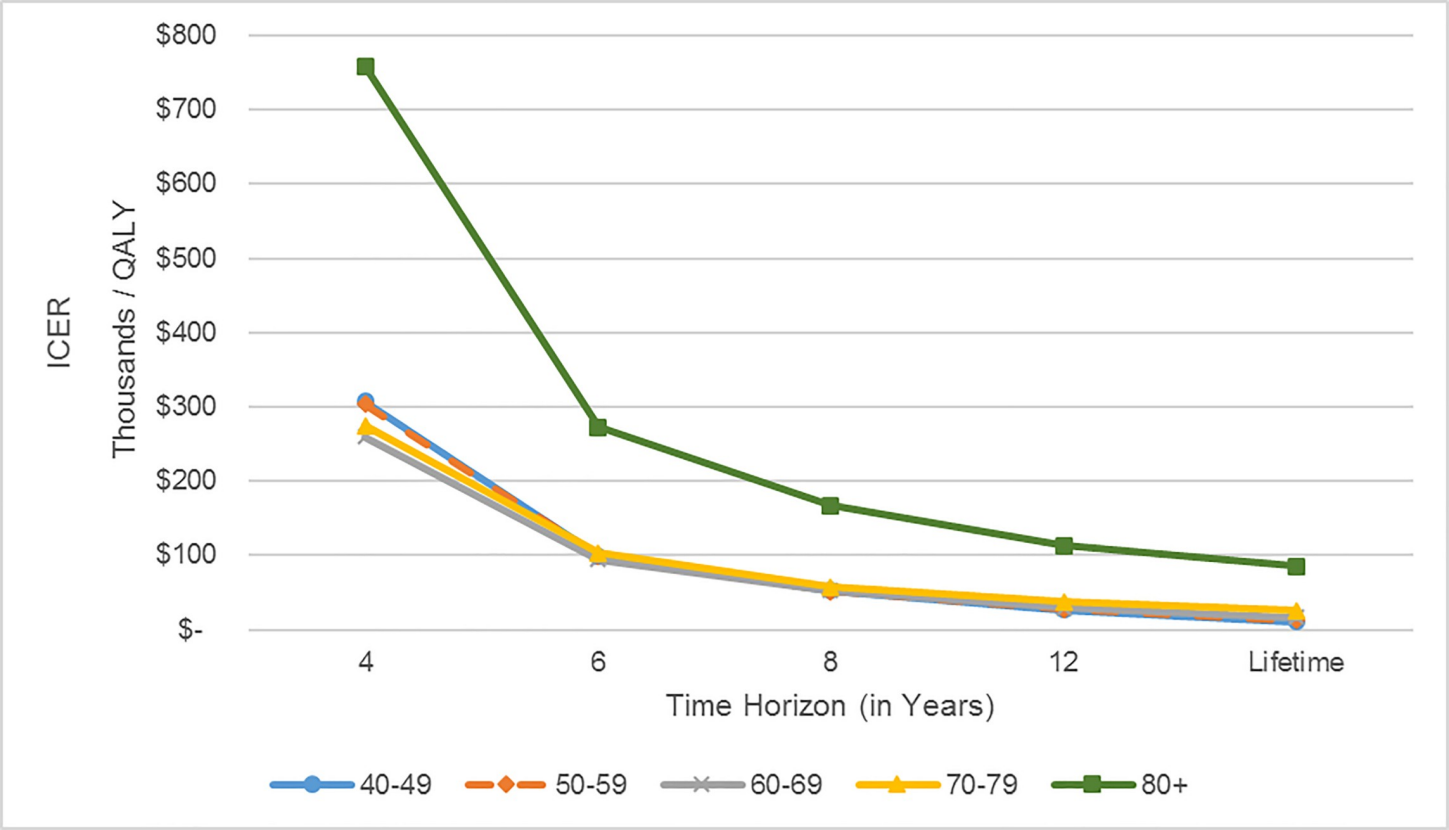
Impact of time horizon on the ICERs ($/QALY) for the TH vs. NT for different age groups.

Time horizon has a significant impact on the ICER (Fig 2; see also S3 Fig). The longer the time horizon (i.e., the time that the effect of treatment on reducing relapse continues), the more cost-effective is the TH regimen compared to the NT arm. If the time horizon for TH regimen median follow-up period is four years (i.e., TH regimen has no additional benefit in reducing relapse beyond four years), the regimen becomes not cost-effective. In our base case analysis, we assumed that while the treatments reduce relapse through lifetime, the rate of reduction in relapse probability is smaller after year 4. Across all age groups, the ICER for TH vs NT under lifetime horizon is relatively close to that under a 12-year time horizon. Thus, extending the time horizon beyond 12 years has a relatively small impact on the cost-effectiveness of TH.

The time horizon represents the maximum time that the TH is effective in reducing the probability of relapse, the regimen leads to no reduction in relapse beyond the time horizon. Abbreviations: ICER, incremental cost-effectiveness ratio; NT, no adjuvant trastuzumab; QALY, quality-adjusted life year; TH, adjuvant paclitaxel and trastuzumab regimen.

In the base case analysis and with a WTP threshold of $50,000 per QALY, TH regimen is cost-effective for time horizons of 12 years or longer and for patients aged between 40 and 79. However, gains due to treatment become smaller and the TH regimen becomes less cost-effective as the patient is older. Nonetheless, TH regimen continues to be cheaper and leads to higher QALYs compared to both ACTH and TCH regimens for all age groups (see S4 Fig).

### 3.3 Sensitivity analysis

**Fig 3:**
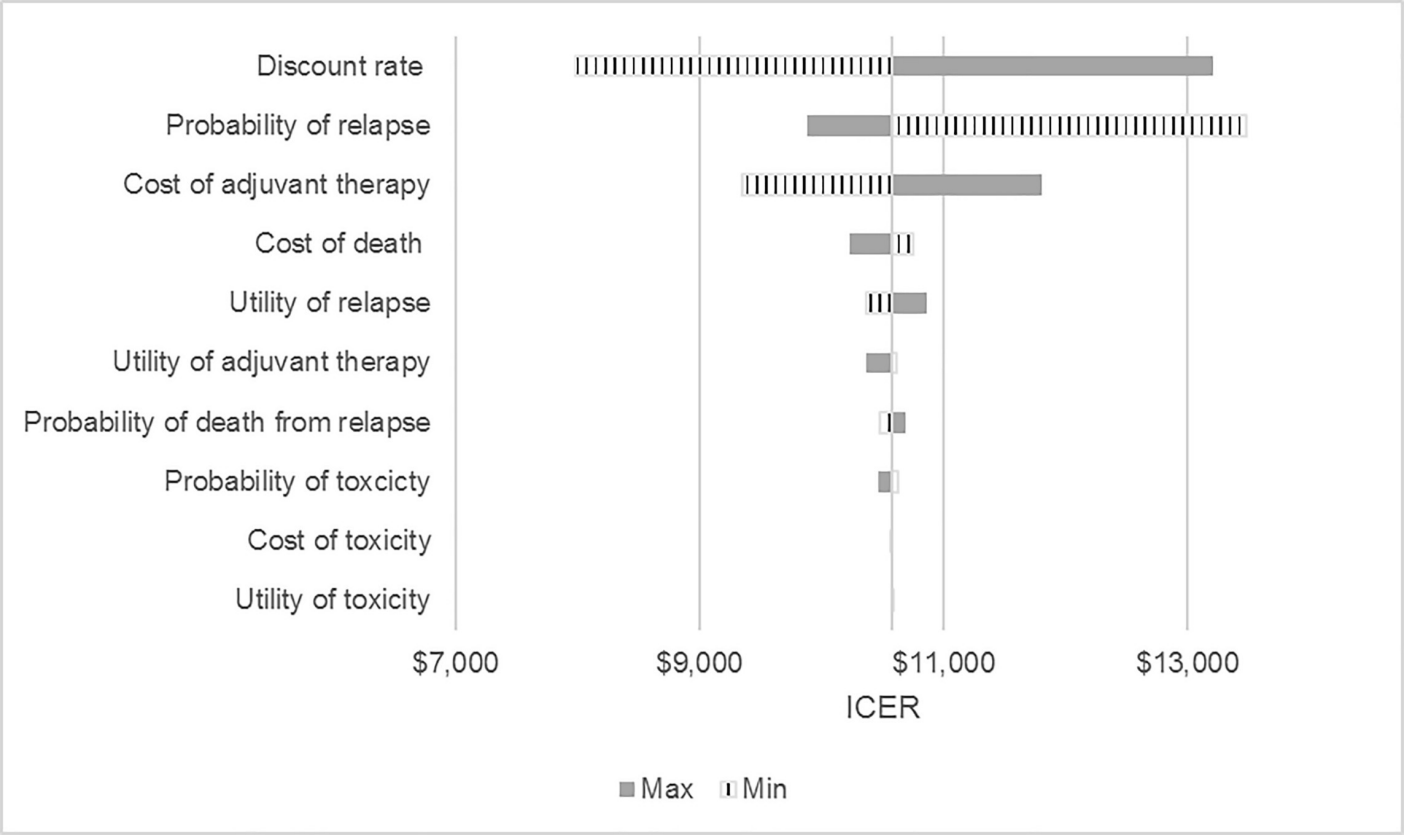
Tornado diagram of one-way sensitivity analyses for the 40–49 age group and lifetime horizon of the TH vs. NT arm. Abbreviations: ICER, incremental cost-effectiveness ratio; NT, no adjuvant trastuzumab; TH, adjuvant paclitaxel and trastuzumab regimen.

We presented the results of one-way sensitivity analysis for the 40–49 age group with lifetime horizon using a tornado diagram (Fig 3). The results are most sensitive to variations in the discount rate, probability of relapse, and cost of adjuvant therapy. Both future costs and QALYs were discounted from 0% to 5% [10,15]. The results are more robust to changes in all toxicity-related parameters, the probability of dying from relapse, and the utility of relapse as well as cost of death.

Our sensitivity analysis on the impact of increasing only the cost of treatment based on the TH regimen shows a very steady increase in the ICER of TH regimen compared to the NT arm; whereas a relatively sharp increase in the ICER compared to both ACTH and TCH regimens (see S5 Fig). At 20% and 50% increase in the cost of TH regimen, the TH regimen is no longer cheaper than TCH and ACTH, respectively. Nevertheless, TH regimen is still cost-effective when compared to all treatment alternatives if the increase in the treatment costs is less than 60%.

The probabilistic sensitivity analysis showed that the likelihood of the cost-effectiveness of TH adjuvant therapy as compared with NT was 100% with a $50,000/QALY threshold (see S1 Table, S6 and S7 Figs).

## 4 Discussion

In this paper, we used a Markov microsimulation model to compare four strategies for treating women with early-stage node-negative, HER2-positive breast cancers. Our base case analysis shows an ICER of $10,584 per QALY for TH compared to NT when we assume that the impact of TH on relapse rates is observed throughout the lifetime of a patient (with a smaller rate of reduction in relapse rates beyond year 4). Our probabilistic sensitivity analysis confirms that the TH regimen is cost-effective in the base case for the 40–49 year–old age group, indicating the robustness of our model and findings. TH regimen is cheaper and leads to higher QALYs compared to both ACTH and TCH regimens for all age groups in the base case scenario as well in the sensitivity analysis due to the relatively high probability of toxicity events and high costs associated with ACTH and TCH.

Several studies reported the cost-effectiveness of widely accepted breast cancer therapies and found favorable ICERs [10,15,20]. For instance, anthracycline-based adjuvant trastuzumab regimen for a 49-year-old women with early-stage HER2-positive breast cancer had an ICER of $39,892/QALY [10], adjuvant trastuzumab chemotherapy for women with HER2-positive breast cancer had an ICER of $26,417/QALY [15], and a more recent study by Hedden et al. reported that the adjuvant trastuzumab chemotherapy for women with HER2-positive breast cancer has an ICER of $13,095/QALY [20].

One-way sensitivity analysis shows that although discount rate, the probability of relapse, and cost of TH are the most influential parameters affecting the ICERs, these parameters still do not have any major impact on the ICERs. For instance, even if the cost of TH regimen is increased by 60%, TH remains cost-effective.

Our study’s major limitation is related to the limited availability of follow-up data on the efficacy of treatment regimens. Namely, our study uses the trial data from the study by Tolaney et al. [7], in which patients were administered with paclitaxel and trastuzumab for 12 weeks, followed by trastuzumab alone for nine months with a median follow-up time of four years. Hence, our model uses this four years of follow-up data for the regimen’s efficacy and toxicity and extrapolates these estimates to the further years. Since the effects of adjuvant trastuzumab persist in reducing the risk of relapse even beyond the completion of adjuvant therapy, we have assumed in our analysis continuing effect of adjuvant therapy with paclitaxel and trastuzumab beyond four years [21,22]. A very recent publication using the longer 7 year follow up data [23] confirms that the trends observed in the study by Tolaney et al. still holds, alleviating concerns about the short-term follow up period [7]. Conversely, if we limit the time horizon of the analysis to four years as used in this trial, TH regimen rapidly loses its cost-effectiveness. To overcome this limitation, we did a sensitivity analysis on the impact of time horizon used in this study and found that the regimen is still cost-effective (with an ICER less than $100,000/QALY) with a time horizon of six years or longer. Moreover, although the majority of the patients in our data sources had a small tumor, some patients with large tumors were included, therefore our definition of early-stage cancer is not limited to a particular size cut off in our analysis. Similarly, our data sources make estimates using small sample sizes for node negative tumors therefore estimates on recurrence may not be accurate. Furthermore, this study uses trial data collected from an academic setting, therefore translation into the real-life situation may not generate the same outcomes. Additionally, our current work utilizes cost estimates from different studies conducted in different contexts. Our future work could include conducting a micro-costing study of real financial data for more accurate estimation. Nonetheless, our sensitivity analysis shows that the cost-effectiveness of TH for treating women with early-stage node-negative, HER2-positive breast cancer is not highly sensitive to costs.

The discovery of trastuzumab has revolutionized the treatment of HER2-positive breast cancer with significant improvements in disease-free and overall survival. However, this does come at a significant cost to the society. With excellent outcomes of low-risk HER2-positive early breast cancer, de-escalation of therapy has generated interest both from an individual patient perspective as well as the society as a whole. This has led to efforts to reduce adjuvant therapies and the evolution of regimens like TH which we hereby show to be cost-effective with an ICER of $10,584 per QALY compared to NT much below the $50,000 per QALY, a commonly used benchmark for cost-effectiveness [24–27]. Although, one year of trastuzumab continues to be the standard of care adjuvant therapy, further efforts are needed to identify potential biomarkers for potential de-escalation of therapy and reduction in cost and toxicities.

In conclusion, based on the current data, the TH regimen has a low relapse risk and improved overall survival in early-stage node-negative, HER2-positive breast cancer, alongside being cost-effective compared to regimens like ACTH and TCH. While awaiting long-term survival data, this regimen is increasingly being considered in clinical use.

## 5 Conclusions

Adjuvant paclitaxel and trastuzumab (TH) is cost-effective for women with early-stage node-negative, HER2-positive breast cancer aged between 40 and 79 years old in the base case scenario as long as the time horizon is longer than the 12 years. Similarly, TH remains cost-effective for women aged between 40 and 49 in the sensitivity analysis. Moreover, TH is cheaper and leads to higher QALYs compared to both ACTH and TCH regimens for all age groups, therefore clinicians should consider utilizing TH for treating women with early-stage node-negative, HER2-positive breast cancer due to its effectiveness and low toxicity.

## Supporting information

S1 Checklist Consolidated health economic evaluation reporting standards (CHEERS) checklist. (DOCX)

S1 Table Probabilistic sensitivity analysis of TH and NT therapies. The table shows the distribution of cost in dollars, QALYs, and ICERs.
(DOCX)

S1 Fig Detailed state transition model. (TIF)

S2 Fig Decision tree representing the markov model for breast cancer treatment. The square represents the decision node, yellow circles represent chance nodes, the purple circles represent Markov nodes, and the green triangles represent the outcome of each random event.
(TIFF)

S3 Fig Impact of age groups on the ICERs ($/QALY) for the TH vs. NT for different age groups. (TIFF)

S4 Fig Scatter plot of incremental cost-effectiveness ratios (ICERs) in thousand dollars ($K) per quality-adjusted life-year (QALY) based on lifetime horizon. (A) Age group 40–49. (B) Age group 50–59. (C) Age group 60–69. (D) Age group 70–79. (E) Age group 80 and older.
(TIF)

S5 Fig Sensitivity analysis on the cost of the TH for the 40–49 age group when lifetime horizon is used. (TIFF)

S6 Fig Probabilistic sensitivity analysis of TH versus NT therapy. Scatter plot of incremental costs (in thousand dollars) and QALYs based on 10,000 samples.
(TIFF)

S7 Fig Cost-effectiveness acceptability curve. (TIFF)
