# Supplementary material for: Cost-effectiveness of adjuvant paclitaxel and trastuzumab for early-stage node-negative, HER2-positive breast cancer
Source: PLoS One. 2019 Jun 5;14(6):e0217778. doi: 10.1371/journal.pone.0217778 (PMC6550431; doi:10.1371/journal.pone.0217778)
Supplement: S1 Table — The table shows the distribution of cost in dollars, QALYs, and ICERs. (DOCX) [file pone.0217778.s002.docx]

| Distribution | TH QALYs | TH Cost | TH LYs | NT QALYs | NT Cost | NT LYs | ICER |
| --- | --- | --- | --- | --- | --- | --- | --- |
| Mean | 16.3 | $ 179,643 | 33.9 | 9.7 | $ 110,744 | 19.2 | $ 10,574 |
| Standard deviation | 0.7 | $ 7,932 | 0.2 | 0.5 | $ 4,989 | 0.6 | $ 2,000 |
| Minimum | 14.5 | $ 157,823 | 33.3 | 8.3 | $ 95,615 | 16.9 | $ 5,279 |
| 2.5% percentile | 15.0 | $ 164,604 | 33.6 | 8.8 | $ 101,114 | 18.1 | $ 7,171 |
| Median | 16.3 | $ 179,660 | 33.9 | 9.7 | $ 110,786 | 19.1 | $ 10,409 |
| 97.5% percentile | 17.7 | $ 194,712 | 34.2 | 10.6 | $ 120,470 | 20.4 | $ 14,923 |
| Maximum | 18.2 | $ 200,152 | 34.5 | 11.4 | $ 126,989 | 21.4 | $ 21,162 |

Abbreviations: ICER, incremental cost-effectiveness ratio; NT, no adjuvant trastuzumab regimen; QALY, quality-adjusted life year; TH, adjuvant paclitaxel and trastuzumab regimen.
