# Supplementary figures and images for: Cost-effectiveness of adjuvant paclitaxel and trastuzumab for early-stage node-negative, HER2-positive breast cancer
Source: PLoS One. 2019 Jun 5;14(6):e0217778. doi: 10.1371/journal.pone.0217778 (PMC6550431; doi:10.1371/journal.pone.0217778)

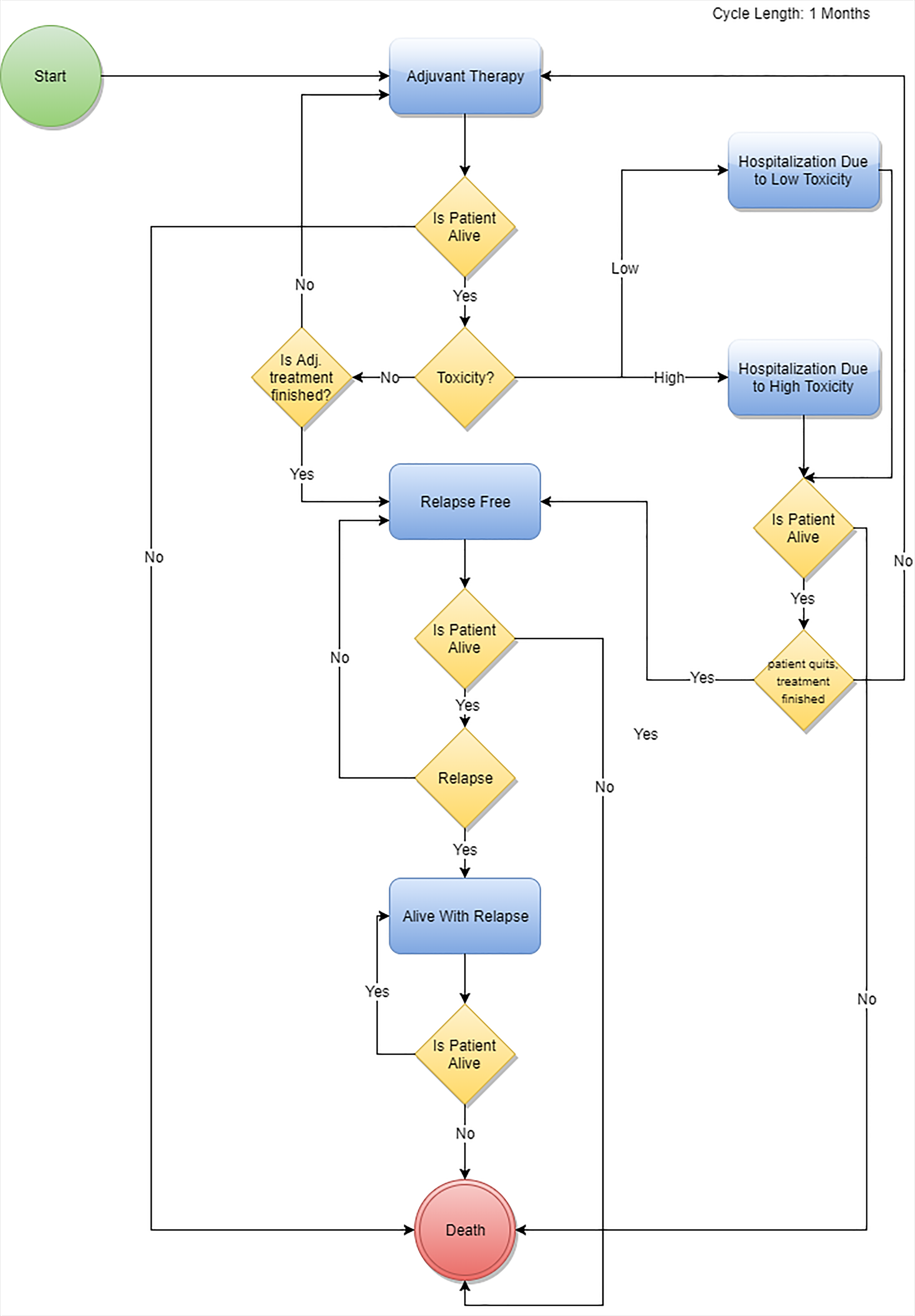

Supplement: S1 Fig — (TIF) [file pone.0217778.s003.tif]

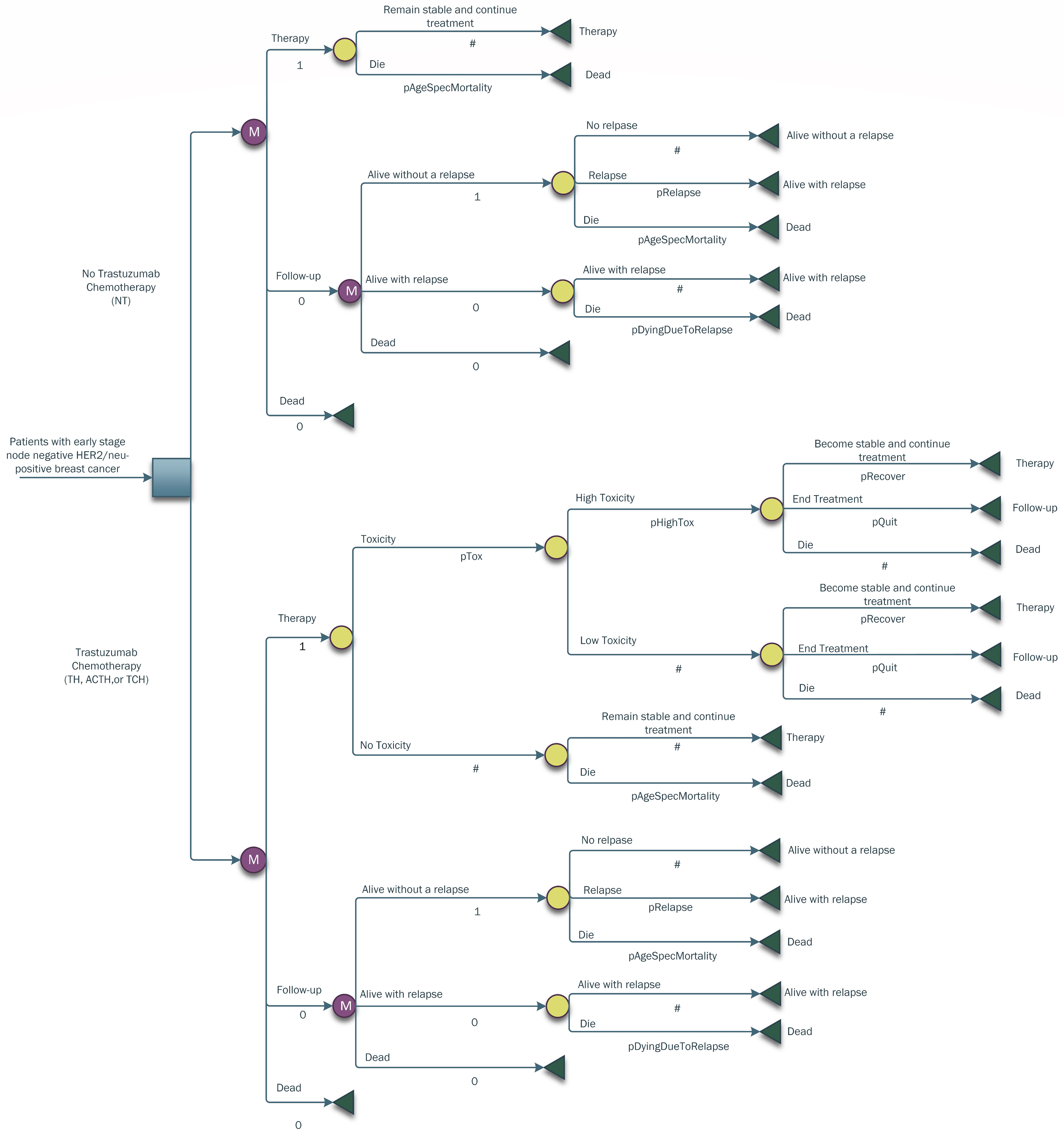

Supplement: S2 Fig — The square represents the decision node, yellow circles represent chance nodes, the purple circles represent Markov nodes, and the green triangles represent the outcome of each random event. (TIFF) [file pone.0217778.s004.tiff]

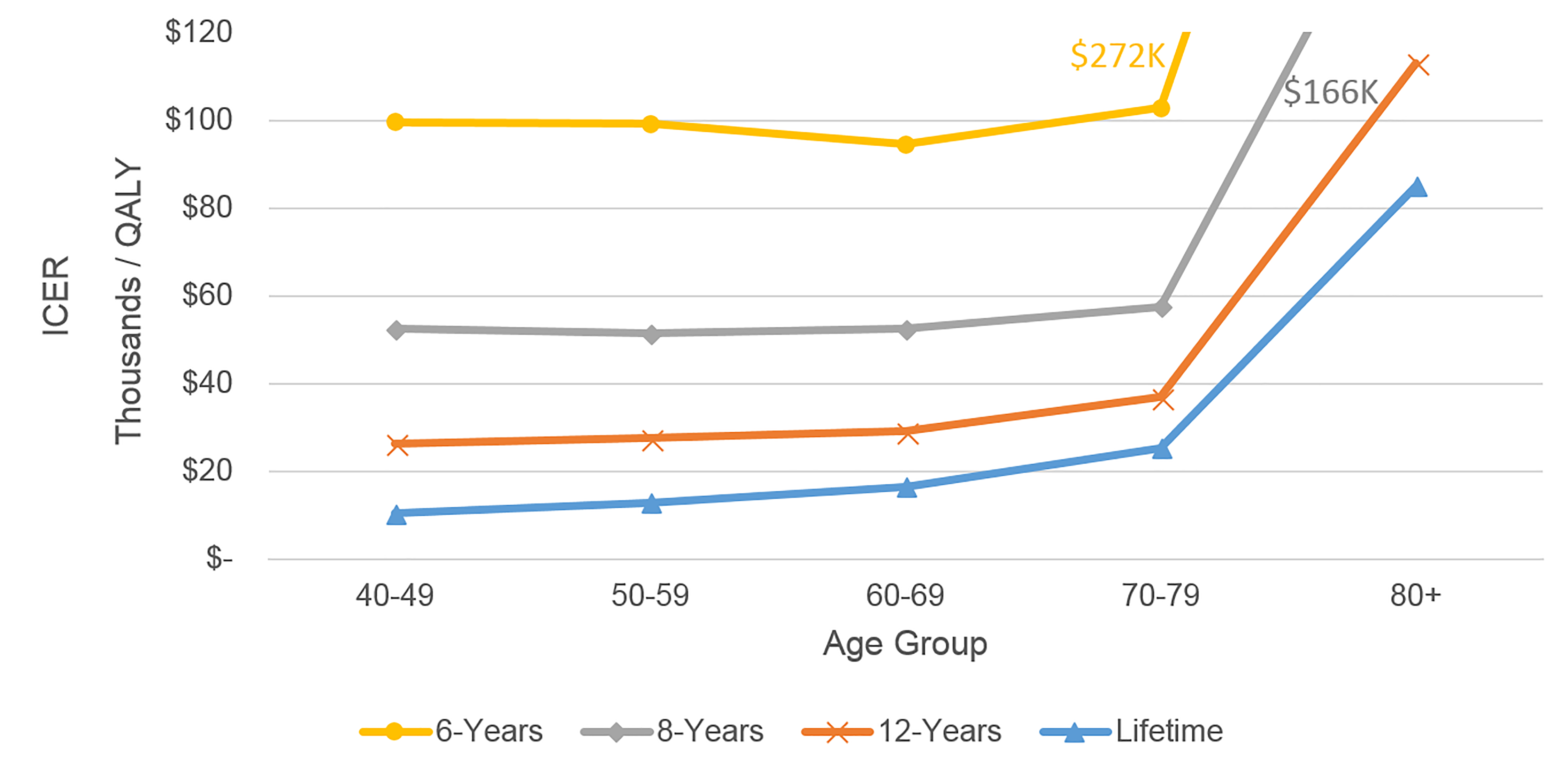

Supplement: S3 Fig — (TIFF) [file pone.0217778.s005.tiff]

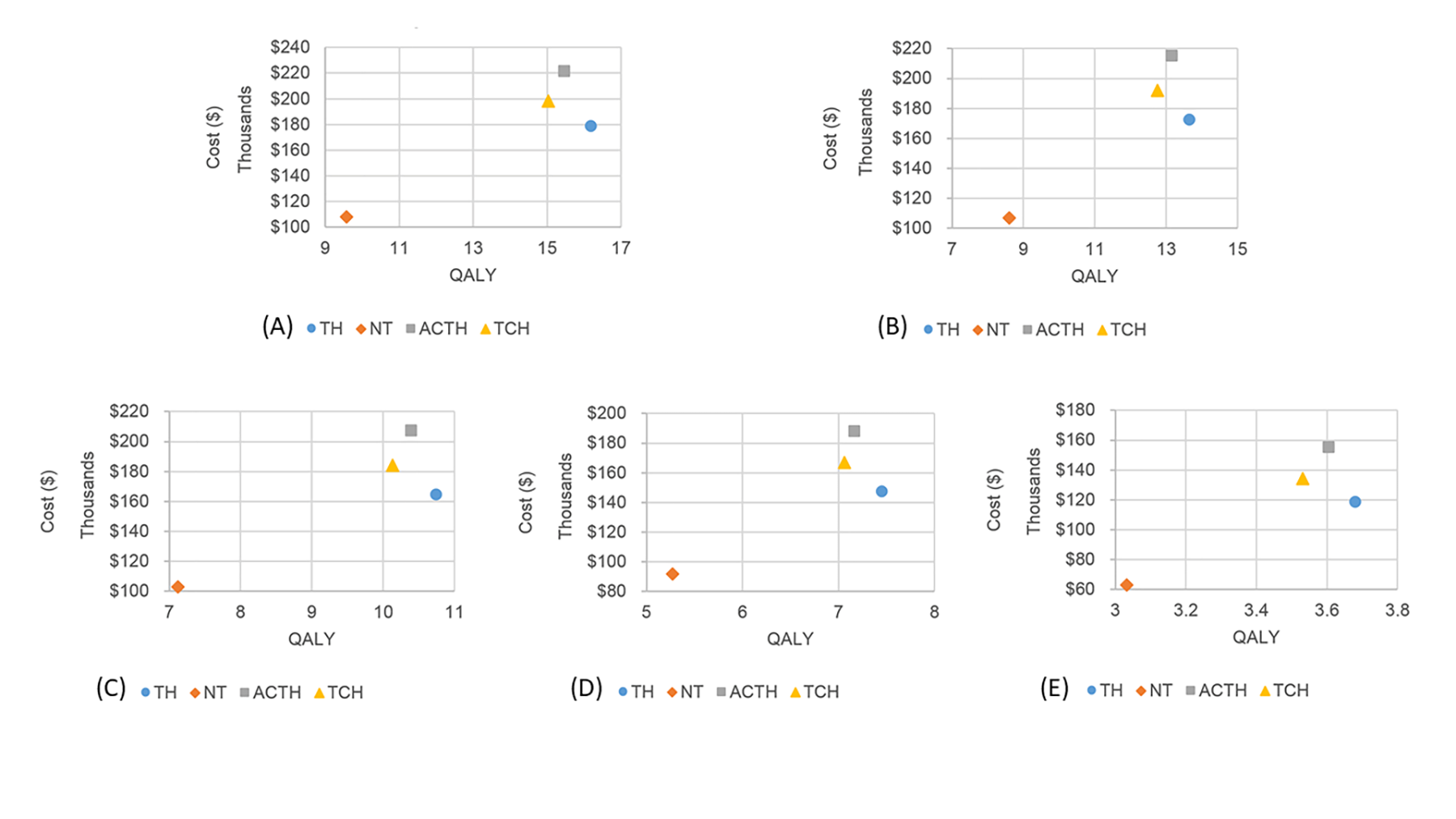

Supplement: S4 Fig — (A) Age group 40–49. (B) Age group 50–59. (C) Age group 60–69. (D) Age group 70–79. (E) Age group 80 and older. (TIF) [file pone.0217778.s006.tif]

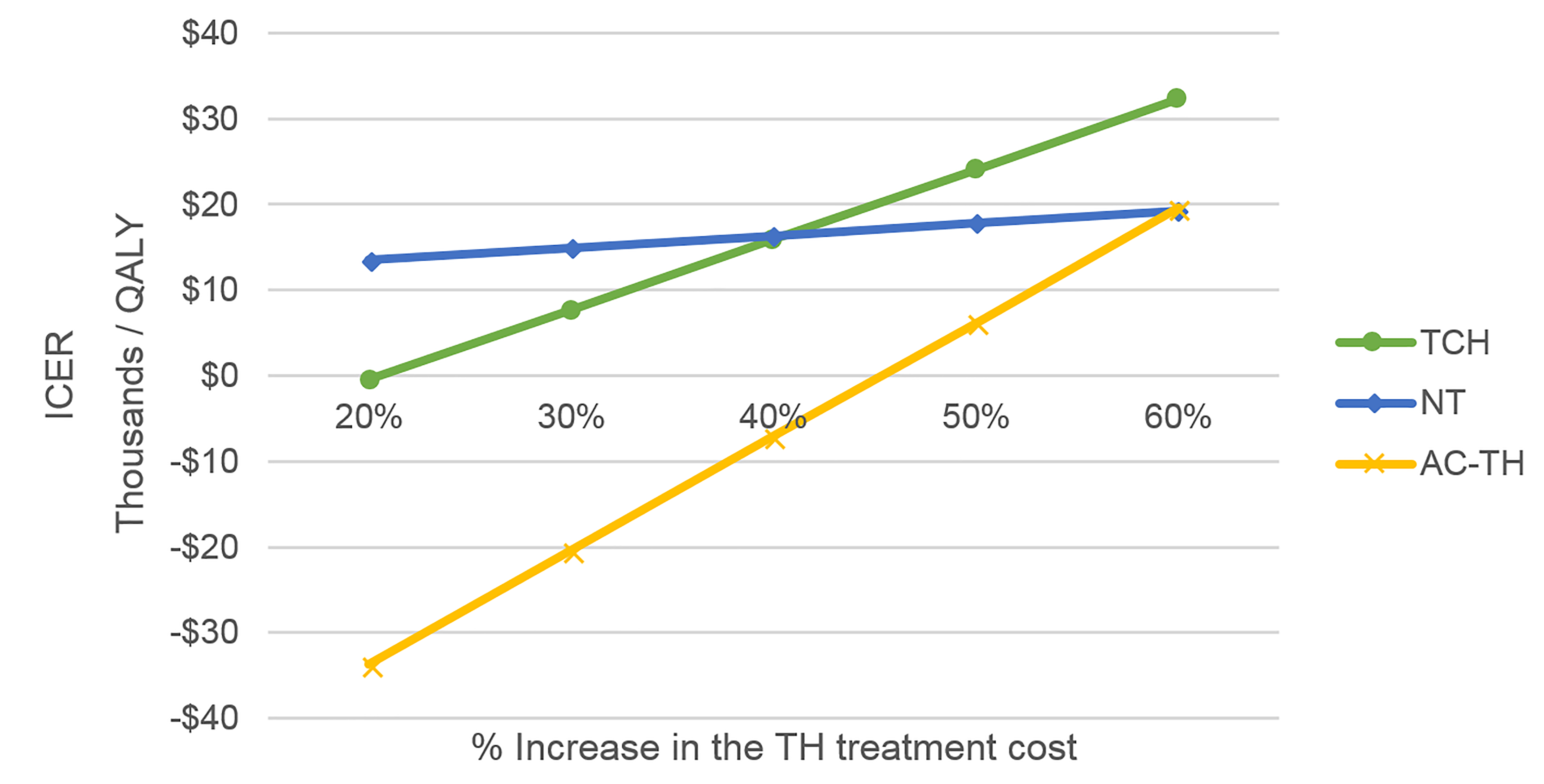

Supplement: S5 Fig — (TIFF) [file pone.0217778.s007.tiff]

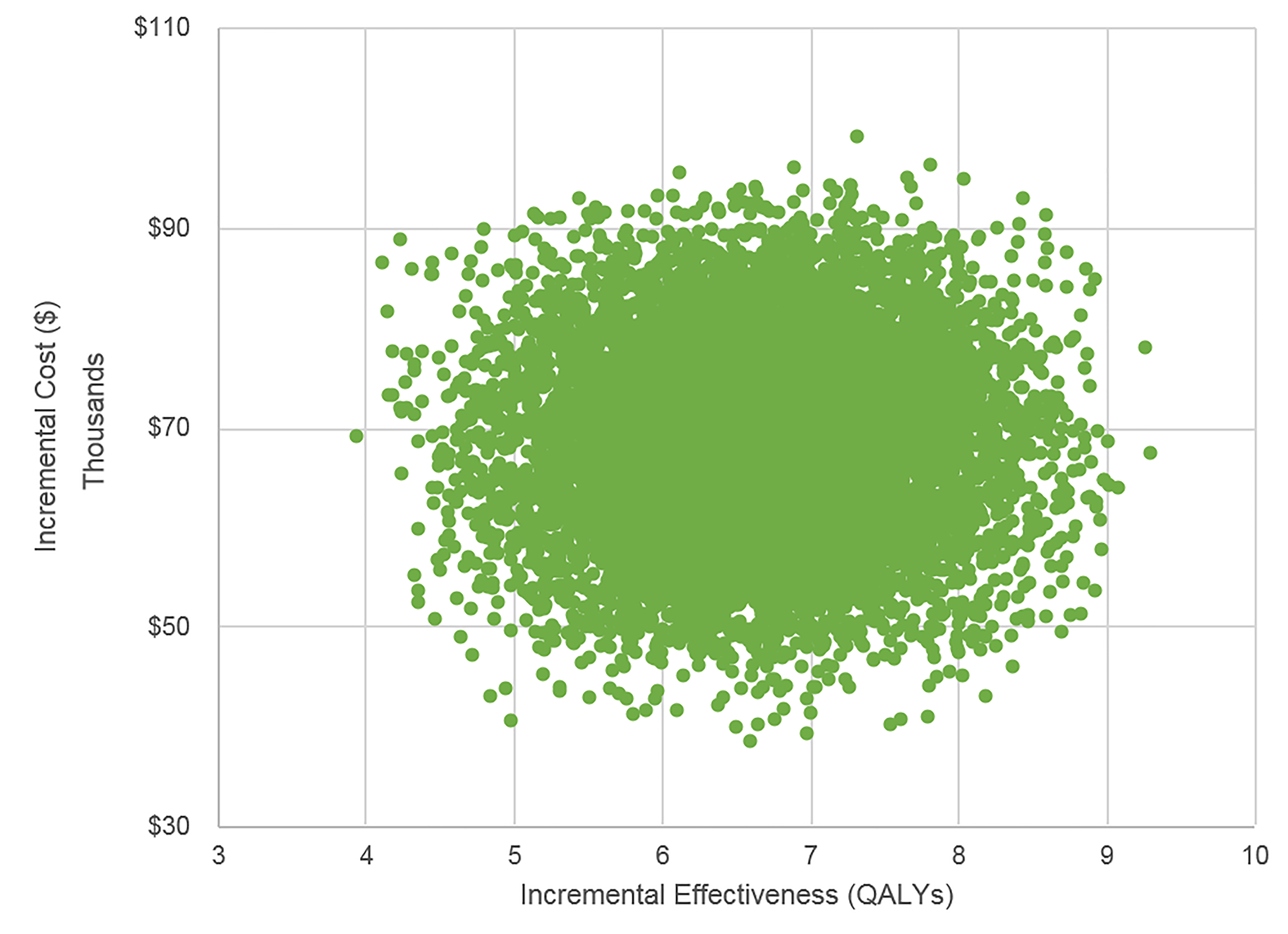

Supplement: S6 Fig — Scatter plot of incremental costs (in thousand dollars) and QALYs based on 10,000 samples. (TIFF) [file pone.0217778.s008.tiff]

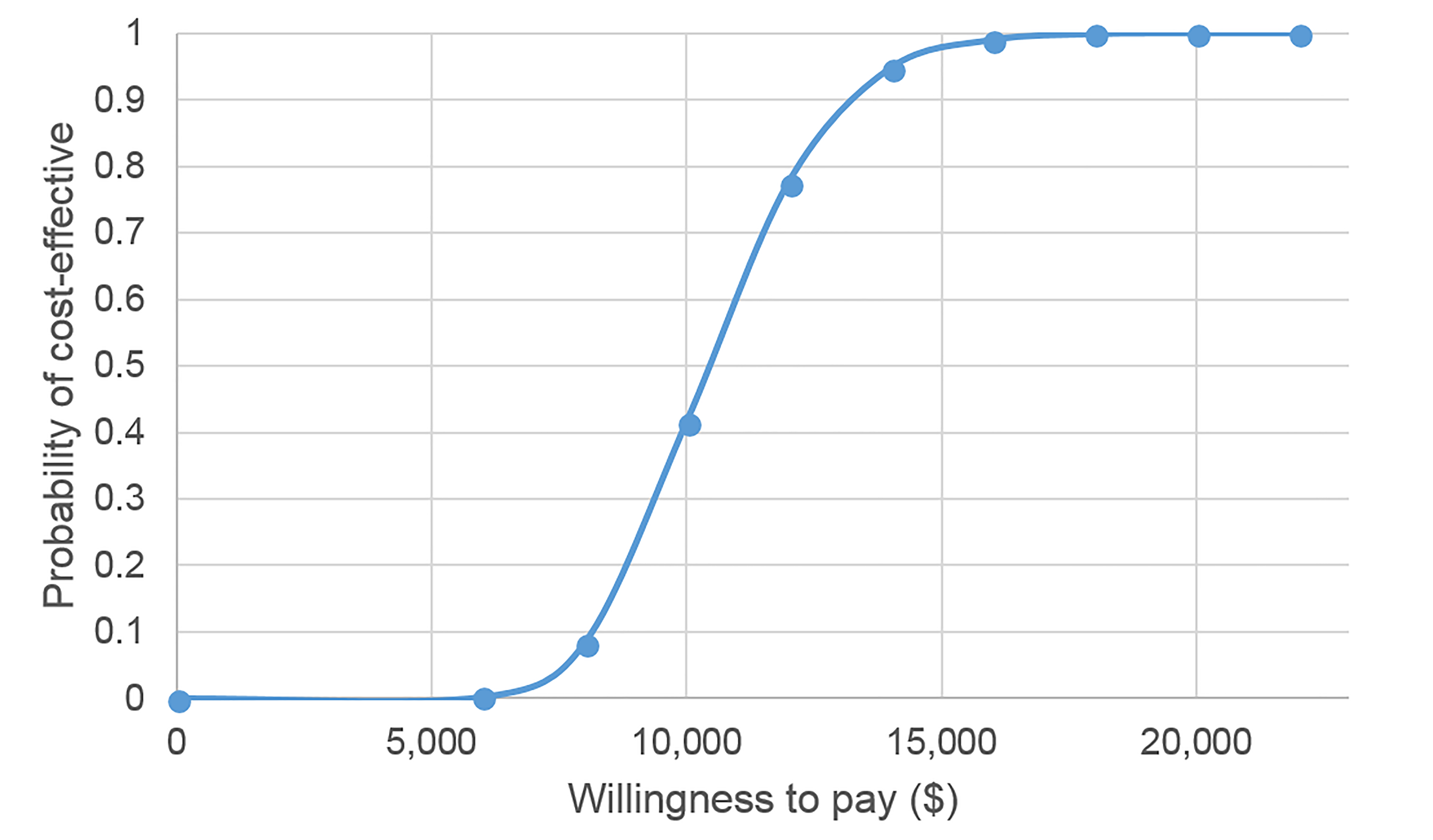

Supplement: S7 Fig — (TIFF) [file pone.0217778.s009.tiff]
